# Correlative studies of a Phase II clinical study of bavituximab and sorafenib in patients with advanced hepatocellular carcinoma

**DOI:** 10.1186/2051-1426-2-S3-P214

**Published:** 2014-11-06

**Authors:** Adam Yopp, Nikoletta Kallinteris, Xianming Huang, Joe Shan, Kerstin Menander, Jeff Hutchins, Steve King, Xiaowei Xu, Dmitry Gabrilovich, Rolf Brekken

**Affiliations:** 1 Department of Surgery, Division of Surgical Oncology, UT Southwestern Medical Center, Dallas, TX, USA; 2 Department of Clinical Affairs, Peregrine Pharmaceuticals Inc., Tustin, CA, USA; 3 Department of Pharmacology, UT Southwestern Medical Center, Dallas, TX, USA; 4 Department of Pathology and Laboratory Medicine, UPENN, Philadelphia, PA, USA; 5 Department of Immunology, Wistar Institute, Philadelphia, PA, USA

## Introduction

Hepatocellular carcinoma (HCC) is the third leading cause of cancer worldwide. The incidence and mortality of HCC have increased three-fold in the United States over the past few years and the majority of patients present with advanced disease. Bavituximab is a novel chimeric IgG1 monoclonal antibody that selectively blocks phosphatidylserine (PS), a membrane phospholipid exposed on the tumor vasculature, tumor cells and tumor-derived exosomes. PS is tightly segregated to the inner leaflet of the plasma membrane, but becomes externalized on the outer surface of dying cells and vascular endothelium in response to oxidative stress, hypoxia, chemotherapy, radiation and other physiological stressors in the tumor microenvironment.

Pre-clinical data demonstrate that Sorafenib increases PS exposure on vascular endothelium and HCC tumor cells. The combination of Sorafenib with a murine Bavituximab analogue potentially inhibited HCC tumor xenograft growth and induced an immunostimulatory macrophage phenotype. Following the preclinical efforts, a Phase I study was completed concluding that Sorafenib (400 mg) and Bavituximab (3 mg/kg) can be safely given in patients with advanced HCC.

## Methods

Patients in an ongoing open-label, single-center Phase II therapeutic study of Sorafenib and Bavituximab, patients consented to undergo two image guided core needle biopsies of a single site of HCC obtained at the time of diagnosis and following one cycle of treatment with Bavituximab and Sorafenib. Treatment cycles consisting of four weekly doses of Bavituximab (3 mg/kg I.V.) and four weeks of Sorafenib (400 mg P.O. BID) were repeated until progression or toxicity. Histologic analysis of immune and myeloid infiltrates (specifically helper and cytotoxic T cells, and macrophages) in tumor tissues was performed.

## Results

In 2 (33%) of the analyzed 6 patients, treatment of HCC patients with Bavituximab and Sorafenib increased infiltration of CD4+ (T-helper cells), and CD8+ (cytotoxic T-cells) ≥ 2-fold, with a corresponding decrease in FoxP3+ (regulatory T-cells) in the tumor microenvironment one cycle post- treatment as compared to baseline. Increased infiltration of tumor associated macrophages ≥ 2-fold was also observed post treatment in these two individuals.

## Preliminary conclusions

Immunohistochemical evaluation of HCC tumor tissues post combination treatment indicated an increase of immune infiltrates; raising the potential of a clinically meaningful anti-tumor immune response. The prevalence of such immune cells within the tumor microenvironment correlates with the pre-clinical experience with Bavituximab in combination with Sorafenib in murine models of HCC.

